# The Perception of Cultural Ecosystem Services by Tourists in Brazilian Protected Areas

**DOI:** 10.1002/ece3.72469

**Published:** 2025-11-14

**Authors:** Marcela de Frias Barreto, Rodrigo Lima Massara, Arthur Soares Fernandes, Adriano Pereira Paglia

**Affiliations:** ^1^ Laboratory of Ecology and Conservation, Department of Genetics, Ecology and Evolution, Institute of Sciences Biological Sciences Federal University of Minas Gerais Belo Horizonte Brazil; ^2^ Institute SerraDical Research and Conservation Center Belo Horizonte Brazil

**Keywords:** big data, culturomics, human–nature interactions, nature tourism, outdoor activities, sentiment analysis

## Abstract

Protected areas play a crucial role in biodiversity conservation, nature tourism, and the provision of ecosystem services, with cultural ecosystem services shaping key human‐nature interactions. Understanding how visitors perceive these protected areas, whether positively or negatively, is essential for supporting their conservation. This study investigates tourists' perceptions of cultural ecosystem services in 27 Strictly Protected Areas in southeastern Brazil, based on TripAdvisor reviews. Nouns were categorized into four main aspects: structural (physical attributes), aesthetic (nature experiences), sociocultural (culture, religiosity, education) and recreational (leisure activities). Adjectives were classified as positive or negative. Positive adjectives were predominantly associated with all aspects, particularly aesthetics and structure. Structural aspects were more frequently associated with negative descriptors, while aesthetic and sociocultural aspects received fewer negative associations than expected by chance. Additionally, sociocultural and recreational aspects also received little attention. These results suggest that tourists are mainly attracted to the natural aesthetic qualities of protected areas, followed by structural features. This positive relationship between tourists and nature has implications for strengthening conservation efforts, emphasizing the value of natural beauty and well‐maintained infrastructure. Furthermore, incorporating sociocultural elements into management practices could foster stronger collaboration between managers and local communities, promoting a more inclusive and sustainable approach to protected area management. Ultimately, the study highlights the importance of integrating natural, structural, and cultural elements into conservation strategies to improve both ecological preservation and local community involvement.

## Graphical abstract

Understanding both the positive and negative interactions between humans and nature is essential, as they directly influence public support for conservation in protected areas. Our results show that visitors particularly value natural beauty and well‐developed infrastructure, while sociocultural and recreational aspects receive less attention. These findings highlight a generally positive relationship between visitors and nature but also emphasize the need to better integrate sociocultural dimensions to foster community engagement and promote sustainable management in protected areas.

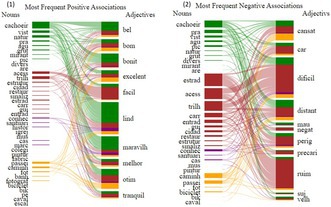

## 1 Introduction

Protected areas (PAs) are pivotal instruments for safeguarding global biodiversity and natural resources (Bruner et al. 2001; Knip et al. 2012; Sánchez‐Azofeifa et al. 1999). Despite their significance, these areas face numerous human‐induced pressures and conflicts, necessitating thoughtful conservation strategies to mitigate such impacts (Chan et al. 2007). Additionally, the conservation of PAs is challenged by high management costs and insufficient resources (Craigie and Pressey 2022; Di Minin et al. 2015). However, PAs are also highly effective in promoting ecosystem services (Eastwood et al. 2016; Zeng et al. 2020).

Ecosystem services are the benefits that ecosystems provide to humans (MEA 2005). They are classified into provisioning, regulating, supporting, and cultural services, with cultural services being particularly relevant to human–nature interactions and relations. Cultural ecosystem services (CES) encompass the intangible benefits individuals derive from ecosystems, influencing their physical and mental well‐being (Haines‐Young and Potschin 2013). A sense of place, considered a CES, refers to the emotional and symbolic connections that individuals develop with specific environments, which can influence their attitudes and behaviors towards conservation (Hausmann et al. 2016). These services play a role in enhancing human health, contributing to physical and psychological well‐being (Daniel et al. 2012). Nature engagement, for instance, has been linked to improved cardiovascular function and increased longevity among the elderly (Takano et al. 2002; Tsao et al. 2022), enhanced learning (Russell et al. 2013) and reduced stress and negative emotions. Despite their substantial value to individuals, CES pose challenges in terms of characterization and measurement (Chan et al. 2012; Hirons et al. 2016; Satz et al. 2013).

Tourist visitation in PAs is a prominent example of CES, providing recreational opportunities and shaping human perceptions of natural landscapes. The concept of perception refers to the way an individual observes, understands, interprets and evaluates, in our case, an experience, and is associated with sensory experiences (Bennett 2016). These perceptions are influenced by factors such as gender, age, social context and cultural values, which shape the diversity of experiences within PAs (Hirons et al. 2016; Plieninger et al. 2013). Thus, tourism provides a way of understanding how individuals perceive and value the CES provided by PAs. PAs can evoke both positive and negative feelings among visitors (Terraube et al. 2017). Understanding the different perceptions of individuals visiting PAs, whether positive or negative, is crucial for improving the overall tourist experience and gaining greater support for conservation initiatives (Mccool 2006).

Understanding tourists' perceptions of CES is essential for effective conservation management and planning. Several approaches exist to assess CES (Cheng et al. 2019). However, recent advancements in data science have introduced big data methodologies, enabling the analysis of large‐scale datasets to identify patterns and correlations (Sagiroglu and Sinanc 2013). Big data analysis is increasingly applied in ecology and conservation to assess the distribution of CES (Clemente et al. 2019; Pastur et al. 2016; Retka et al. 2019) and to evaluate landscapes with tourist potential based on user preferences (Bachi et al. 2020). This includes investigating whether charismatic species drive the use of social media (Hausmann et al. 2017) and exploring innovative approaches to enhance recreation in PAs (Sinclair et al. 2022).

Tourist visitation is increasingly being assessed through big data methods, using data from the internet and social media. Notably, studies have explored wildlife tourism, focusing on the animals most frequently associated with such activities (D'Cruze et al. 2018), visitation patterns, common tourist activities, popular destinations (Heikinheimo et al. 2017) and tourists' preferences regarding park biodiversity (Hausmann et al. 2018). Studies also show that different groups of tourists (locals or international tourists) can use an area's CES in different ways (Ghermandi et al. 2020). Other studies aim to identify factors influencing recreational activities and methods for mapping them in landscapes (Nyelele et al. 2023). Additionally, there is an emphasis on analyzing visitors' emotions, including feelings such as joy and surprise, as well as their polarity (positive, negative, and neutral) (Hausmann et al. 2020; Souza et al. 2024).

The advent of data sciences and the widespread availability of large open‐access databases, including social networks, present new opportunities for advancing studies in conservation biology (Di Minin et al. 2015) and tourism research. Text mining and natural language processing (NLP) techniques are increasingly used to evaluate information from texts found in social networks and scientific publications (Toivonen et al. 2019; Westgate et al. 2015). One notable approach employing NLP is sentiment analysis, which involves evaluating the content of a text, including emotions, attitudes, and opinions, often classified as positive, negative, or neutral (Fink et al. 2019). This analysis has been applied in rate CES by a community through opinions gathered in questionnaires (Lee et al. 2020) and to assess the sentiments of tourists visiting parks (Hausmann et al. 2020).

Here, we aimed to deepen our understanding of tourists' perspectives on the CES provided by PAs in state of Minas Gerais, southeastern Brazil. Given that cultural ecosystem services are provided within conservation units and can be perceived by visitors through diverse aspects such as aesthetics, infrastructure, and socio‐cultural and recreational elements, we hypothesize that perceptions of these aspects will be predominantly positive. A large number of positive comments would reflect the physical and psychological benefits these areas offer their visitors. Furthermore, based on the premise that PAs attract visitors seeking closer contact with nature, we expected tourists' TripAdvisor comments to emphasize the aesthetic (i.e., natural) aspects of PAs more frequently and more positively than other aspects. A stronger emphasis on natural attractions may suggest that visitors place particular value on these features and, by extension, support their conservation. By confirming our working hypothesis, we could provide park managers with new management tools, helping them to optimize the cultural ecosystem services offered to visitors.

## 2 Methods

### 2.1 Study Areas and Sampling

To identify suitable areas for this study, we conducted a search for PAs within the state of Minas Gerais. We specifically focused on PAs that include the domains of the Cerrado and Atlantic Forest biomes within their territory, although one of the areas represents a transition between Cerrado and Caatinga. In addition, we considered Brazilian PA categories that legally allow only indirect use of natural resources and permit tourist activities. These categories include Parks, Natural Monuments, Wildlife Refuges, and Private Natural Heritage Reserves (known as Reservas Particulares do Patrimônio Natural—RPPNs in Portuguese). Importantly, all these categories align with IUCN Categories II and III.

**FIGURE 1:**
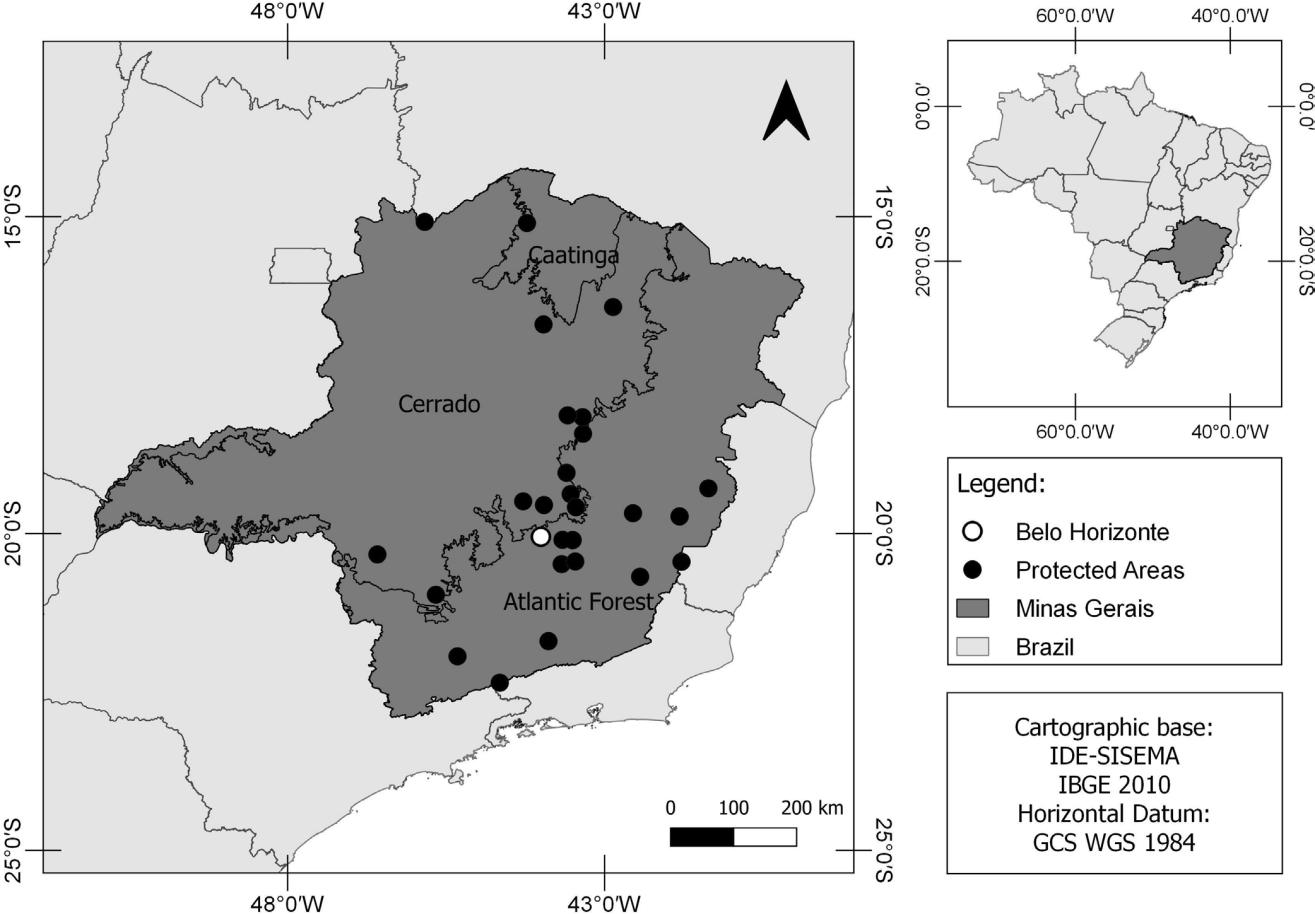
Distribution of 27 Protected Areas with tourists' comment collected for this study in the state of Minas Gerais, southeastern Brazil. The map of Minas Gerais shows the biomes (Atlantic Forest, Cerrado and Caatinga). The white point indicates the Rola Moça State Park, which is partly in Belo Horizonte, the capital of the state.

Subsequently, we conducted searches for these identified PAs on the TripAdvisor website, where we retrieved tourist reviews. TripAdvisor, a specialized platform for tourist information, serves as a hub for tourists to share comments about diverse destinations. We considered exclusively Portuguese‐language comments, which constitute an overwhelming majority of reviews for PAs in Minas Gerais, collected between March 2011 and May 2020. We prioritized Portuguese reviews because automated translations can introduce inconsistencies or distort nuances, potentially compromising the validity of sentiment and text‐mining analyses. By limiting our analysis to Portuguese comments, we ensured consistency in language processing and maintained the integrity of the sentiment classification process. Importantly, the personal identities of the reviewers were never disclosed, as TripAdvisor protects user privacy and our dataset excludes any identifying information. In 2020, these comments were manually collected and systematically stored in a database. The dataset comprised comments from tourists who visited 27 Protected Areas in the state of Minas Gerais (Figure 1), encompassing 24 Parks, one Natural Monument, and two RPPNs (Supporting Information Table A1).

These PAs offer a multitude of attractions, including waterfalls, lakes, lagoons, peaks, wells, caves, and cave paintings. Some also feature religious and historical‐cultural attractions such as museums, chapels, and churches (IEF 2020; MMA 2013; SEMAD et al. 2007a). Leisure activities within these areas range from hiking and trekking to mountain biking and horseback riding (Penido et al. 2005). Notably, the PAs differ greatly in their infrastructure, with some being well‐equipped and offering accommodations (e.g., camping, lodging, houses, etc.), restaurants, snack bars, and visitor centers, among other amenities (IEF 2020; SEMAD et al. 2007b).

### 2.2 Data Analysis

To analyze the text data, we included only nouns and adjectives that appeared more than five times across comments. Following the content analysis framework of Bardin (2018), we grouped words with similar meanings into thematic clusters. Initially, we generated frequency lists of nouns and adjectives from all comments. Then, two authors collaboratively classified each word based on context and dominant usage in the comments, using the free version of Sketch Engine (Kilgarriff et al. 2004, 2014). Subsequently, nouns were categorized into four thematic aspects, adapted from Costanza et al. (1997), focused on CES and nouns that would not be associated with any of the aspects were exempted from the list. This categorization facilitated meaningful comparisons among service categories relevant to the study, as outlined below:

1. **Esthetics (AE):** This category encompasses words related to the interaction with and appreciation of natural attractions and features in a protected area, such as waterfalls, peaks, and landscapes. This classification was emphasized due to its importance in understanding the appreciation of natural elements in the context of this study.
2. **Recreation (RE):** This category includes words associated with tourist activities that natural areas provide, such as hiking and photography, reflecting leisure and eco‐tourism opportunities.
3. **Sociocultural (SO):** This aspect includes words related to the interaction with and appreciation of human cultural elements, excluding esthetics. It incorporates terms linked to cultural traditions, gastronomy, spiritual/religious values (e.g., religion and faith), and education/knowledge (e.g., research and learning). Physical structures tied to these values, such as museums, churches, and libraries, were also included.
4. **Structure (ST):** Although not a cultural ecosystem service per se, this category includes words describing physical and personal infrastructure that influences the perception of these services. Examples include restaurants, toilets, trails, and staff associated with protected areas.

Adjectives were classified into two qualifiers:

1. **Positive (PO):** Includes adjectives such as “good” and “beautiful.”
2. **Negative (NE):** Includes adjectives such as “bad” and “ugly.”

Words categorized as not relevant—such as place names—and those representing multiple aspects (e.g., “atrativo” in Portuguese, which broadly refers to any tourist attraction like a waterfall or museum and may span esthetic, sociocultural, or recreational services), as well as ambiguous qualifiers (e.g., “grande” in Portuguese or “long” in English, which cannot be definitively classified as positive or negative)—were excluded from the analyses.

We performed sentiment analysis to link tourists' sentiments (positive or negative qualifiers) with different categories of the protected area (AE, RE, SO, and ST). Data preprocessing followed fundamental Natural Language Processing techniques and was performed on the full data (comments) and on the previously classified words. The first step involved tokenization, dividing the text into individual words or meaningful elements (tokens) (Gobbo 2019). Tokenization is a crucial step for standardizing the vocabulary, as it treats variations of the same word such as plural forms, different genders, or distinct suffixes as a single entity, which increases the ability of comparison of the units of text analyzed.

Tokens shorter than three characters and stopwords (e.g., pronouns, conjunctions, and numerals) were excluded using the categorized dictionary provided by the Quanteda package (Benoit et al. 2018). Stemming was then applied to reduce words to their root forms (Gobbo 2019), ensuring that variations such as plurals (e.g., “montanha” and “montanhas” in Portuguese, which means “mountains” in English) and gender‐specific forms (e.g., “bela”/“belo” in Portuguese, which means beautiful in English) were unified into a single root. After tokenization and stemming, we organized the comment tokens by phrase, keeping only the root forms.

The initially classified tokens served as semantic filters to categorize the remaining comment tokens. To ensure consistency, we converted the encoding from UTF‐8 (which supports specific Portuguese accents) to ASCII//TRANSLIT, treating accented and unaccented versions of the same word (e.g., “ótimo” and “otimo” in Portuguese, which means great in English) as identical. Tokens belonging to multiple categories after preprocessing were removed. The tokens used in our work, with their respective frequencies and classification, are listed in the Supporting Information (Supporting Information Table A3).

**FIGURE 2:**
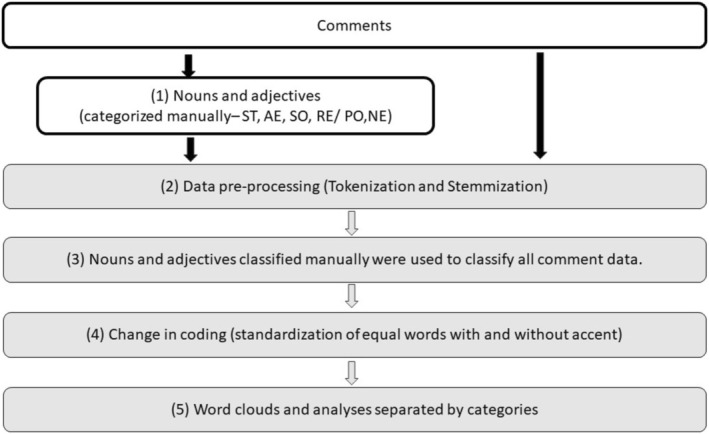
Stages of text processing to create word clouds. Nouns were classified into aspects and adjectives into qualifiers (1). Comments were divided into sentences, and text processing was performed on the entire dataset (2). Aspects and qualifiers served as semantic filters (3). The encoding was adjusted to treat words with and without accents as equivalent (4). Word clouds were generated, and analyses were categorized (5).

Text processing was performed using the R programming language (R Core Team 2025), with the Quanteda package (Benoit et al. 2018). These steps reduced the text corpus to 15% of the original comment tokens. Finally, word clouds were generated using the wordcloud package (Fellows 2018), visually representing the most frequent nouns and adjectives (Figure 2).

To assess the relationship between aspect and qualifier categories, we posited that the co‐occurrence of words harbors latent semantic relations within each specific pairing (Evangelopoulos et al. 2012). Initially, these were structured as bigrams of word interactions to quantify such semantic relations. Importantly, we assumed that qualifiers (adjectives) would carry semantically relevant meaning only when associated with aspects (nouns) in the same sentence. To compare the interactions obtained between qualifiers and aspects, we used a graphical system representation for sentiment analysis (Aisopos et al. 2011). Network analysis tools (Barabási 2016) were then employed to assess the existence of association patterns between aspects (nouns) and qualifiers (adjectives). The resulting interaction network comprised vertices (nouns and adjectives) and links between them. Only links between nouns and adjectives were allowed, and irrelevant links to our study, such as those between two tokens of the same aspect or qualifier, were removed. This type of interaction network, where links are allowed only between groups and not within a group of vertices, is termed bipartite. The “igraph” package (Csardi and Nepusz 2006) in the R programming language was used to construct the network. Using the “ggplot2” package in R, we created graphs to represent the tokens with the highest frequency of interactions. Finally, a contingency table was created to assess the pairwise distribution of associations between adjective and noun classes. Chi‐square tests were employed to determine whether the observed frequencies of these relationships differed from the expected ones (Agresti 2007).

## 3 Results

**FIGURE 3:**
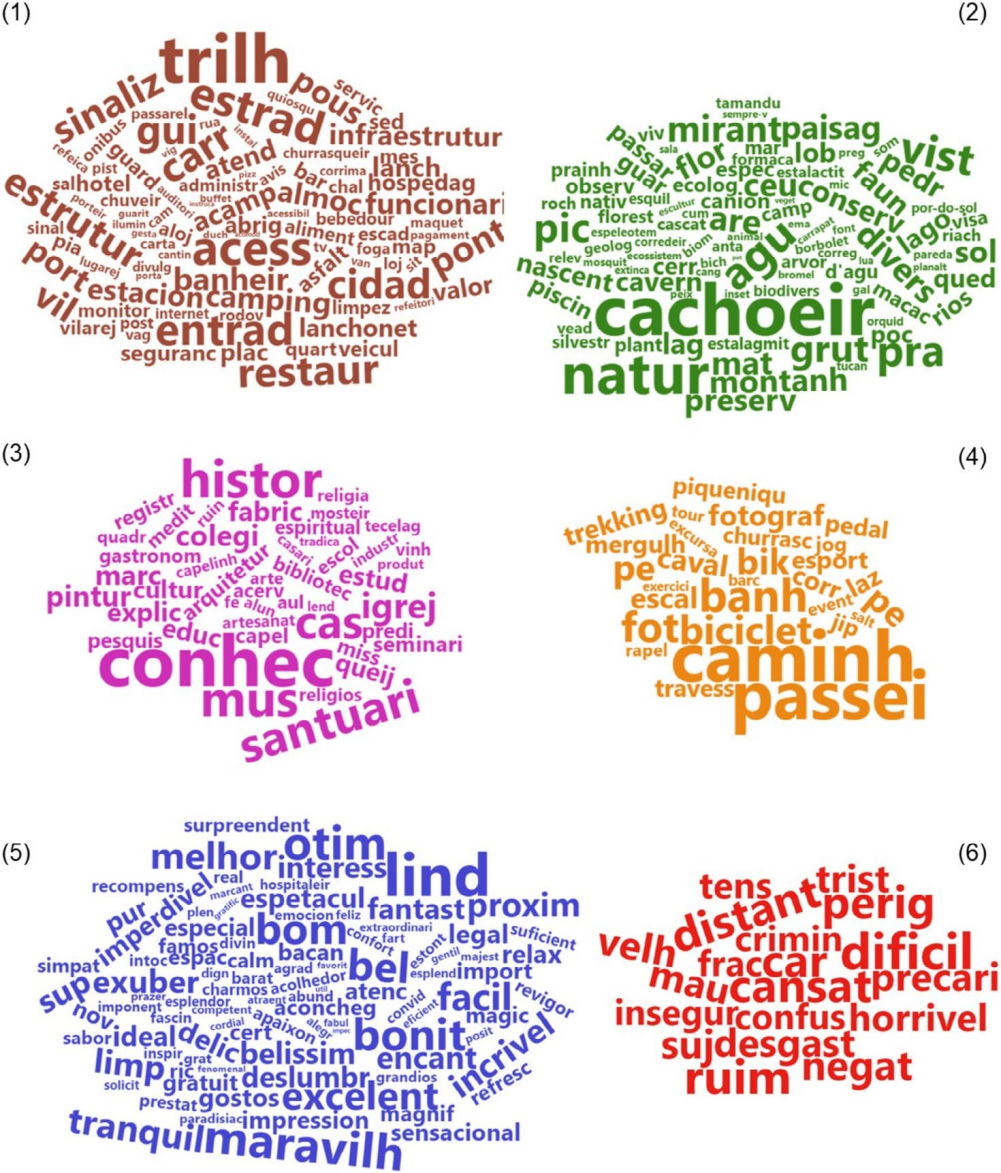
Word clouds for each of the categories. The size of the words indicates the frequency of that word in the comments. The word clouds are: (1) structure, (2) esthetics, (3) sociocultural, (4) recreational, (5) positive, and (6) negative. Some tokens were translated into English (Supporting Information Table A2).

We collected a total of 6537 TripAdvisor comments from tourists who visited the selected PAs, resulting in 8219 sentences. From these, 1509 nouns and adjectives were identified, comprising 443 categorized words. Most adjectives conveyed a positive sentiment, including terms such as “alegre” (joyful), “emocionante” (exciting), “feliz” (happy), and “grato” (grateful), among others. Following text processing, the number of tokens per category was distributed as follows: structure (92), esthetics (95), sociocultural (48), recreational (29), positive (104), and negative (20) (Figure 3). Despite both positive and negative qualifiers being associated with all PA aspects, the overall sentiment expressed by tourists was predominantly positive during their visits to the PAs. Here's a comment from 2017 about Ibitipoca State Park as an example: “Parque muito bonito, belas trilhas, belas cachoeiras, boa infraestrutura na sede do parque, equipe de guardas muito atenciosos”. This sentence shows the person using positive adjectives (English—“nice”, Portuguese—“belas”, token—“bel”) to praise esthetic aspects (English—“waterfalls”, Portuguese—“cachoeiras”, and token—“cachoeir”) and structural aspects (English—“trail”, Portuguese—“trilha”, and token—“trilh”).

**FIGURE 4:**
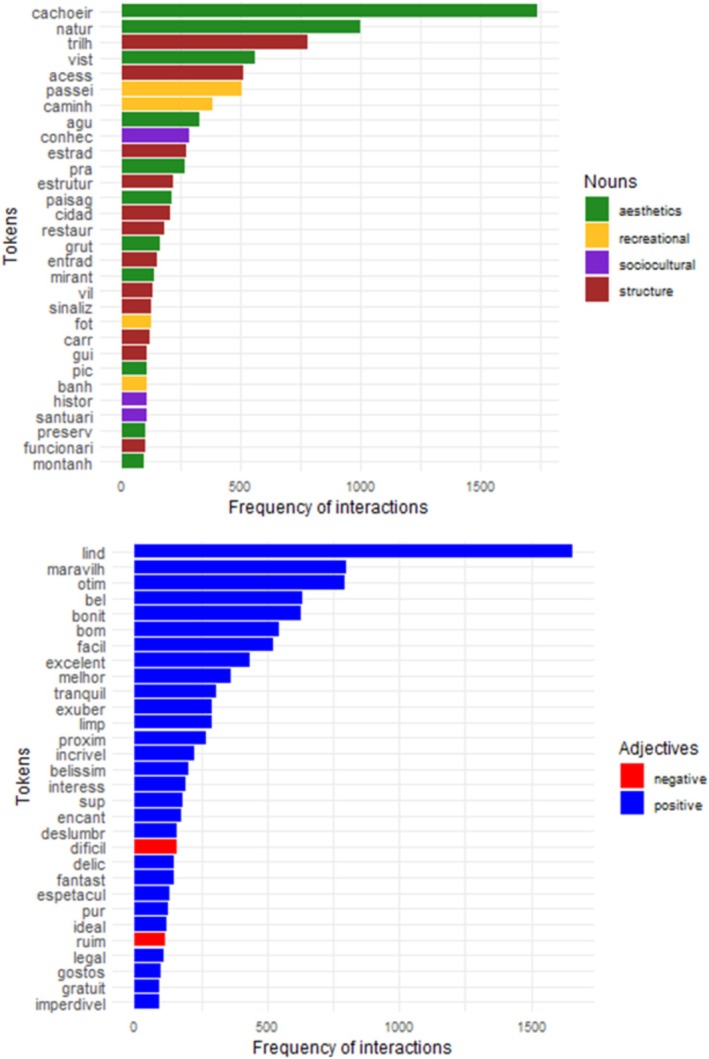
The graphs show the frequency of interaction for each token. Each graph shows the 30 most frequent tokens, with the top graph representing aspects (nouns) and the bottom graph representing qualifiers (adjectives). Some tokens were translated into English (Supporting Information Table A2).

The frequency values for each token provided further insight into the predominance of certain aspects and qualifiers in the comments. Notably, tokens related to esthetic aspects (e.g., “cachoeira”, “natureza”, “vista” in Portuguese, which means “waterfall,” “nature,” “view” in English) and structural aspects (e.g., “trilha,” “acesso” in Portuguese, which means “trail” and “access” in English) were more prominent. Tokens related to recreational aspects (e.g., “passeio,” “caminhada” in Portuguese, which means “tour” and “walking” in English) were also prominent. In addition, there was a notable presence of positive qualifiers (e.g., “lindo,” “maravilhoso” in Portuguese, which means “beautiful” and “wonderful” in English) (Figure 4).

**FIGURE 5:**
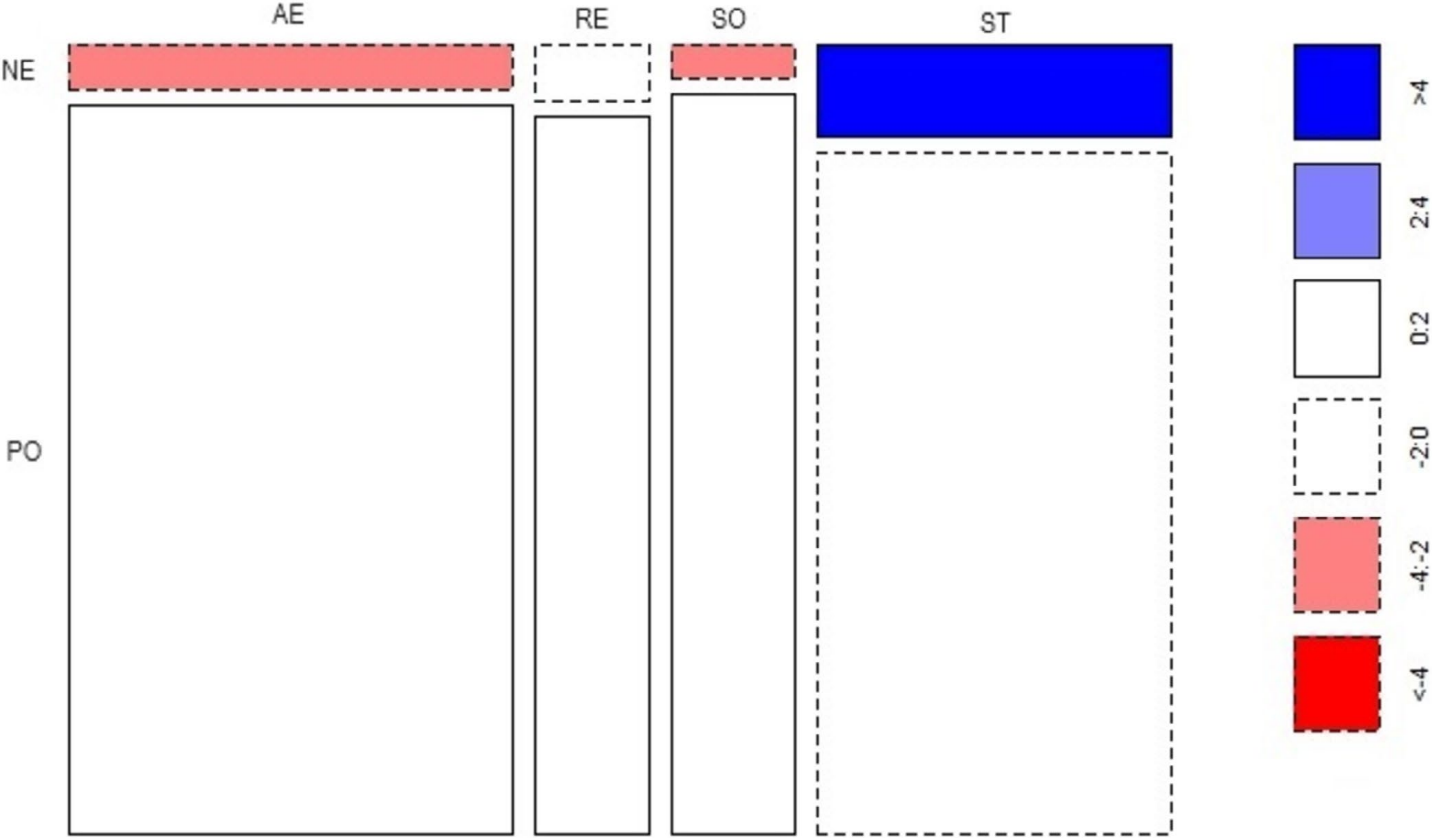
Representation of a chi‐square statistic used to test association between aspect categories (Structure = ST, Esthetics = AE, Sociocultural = SO, Recreational = RE) and sentiment qualifiers (Positive = PO, Negative = NE). This plot visualizes standardized residuals from the chi‐square test, highlighting cells where observed frequencies differ from those expected by chance (*p* < 0.05). Solid outlines mark cells where observed values are significantly higher than expected. Dashed outlines indicate cells where observed values are significantly lower than expected. Colored fills represent statistically significant residuals, with blue for over‐representation and red for under‐representation. Shade intensity reflects the magnitude of deviation—darker colors denote larger residuals. White or unfilled cells denote nonsignificant differences (|residual| < 2).

The relationship between aspects and qualifiers was significantly different from what would be expected by chance (*χ* ^2^ = 32.839, df = 3; *p* = 3.482e‐07). The association of negative qualifiers with structural aspects was greater than expected by chance (shown in blue in Figure 5). In contrast, the association of negative qualifiers with esthetic and sociocultural aspects was lower than expected by chance (shown in pink in Figure 5—pink).

**FIGURE 6:**
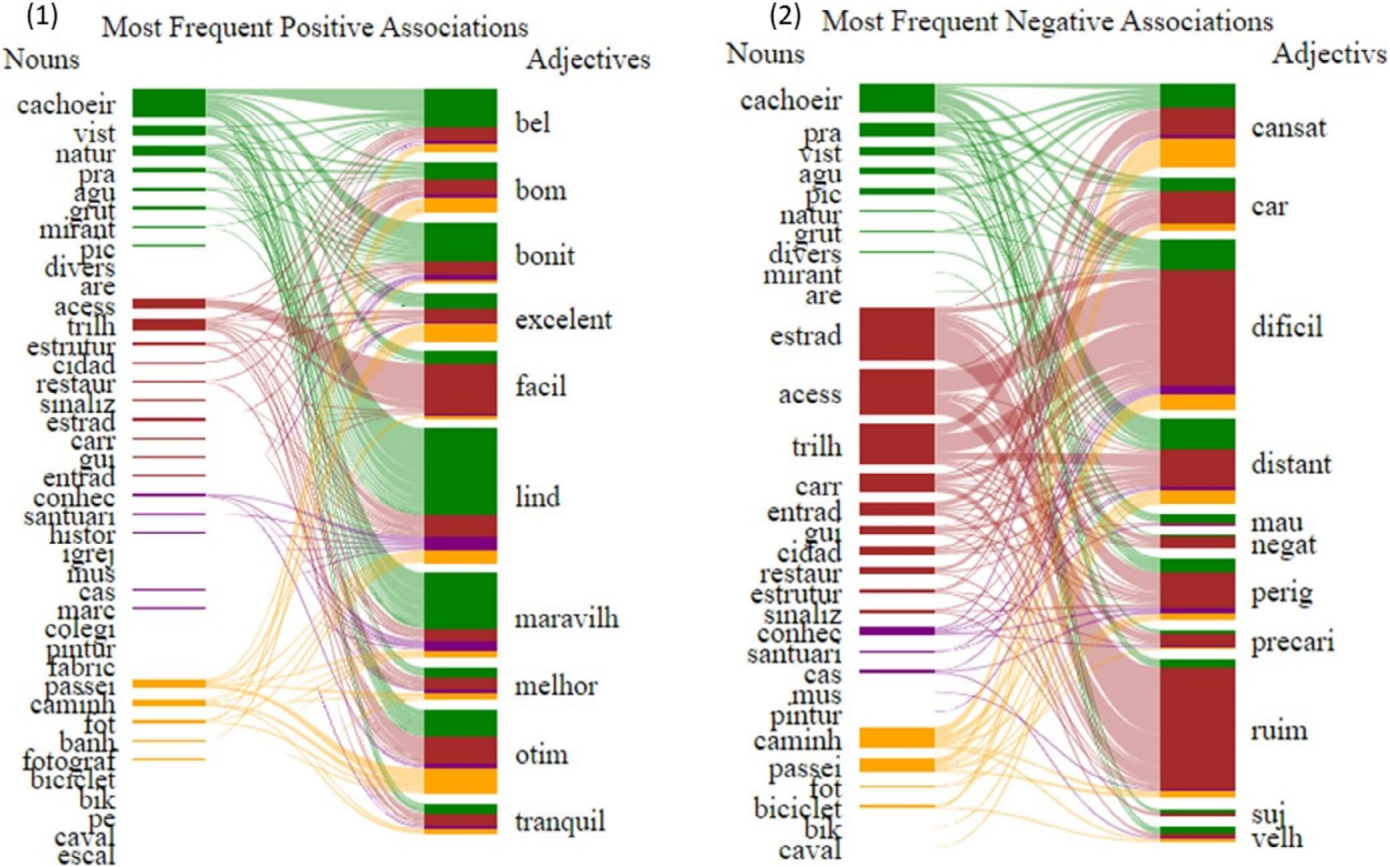
Interaction networks between nouns and adjectives: (1) positive and (2) negative. The frequency of word interaction is shown by the size of the rectangles associated with the tokens. The colors are used to show the associations of each adjective with each aspect: Structure (ST—red), esthetics (AE—green), sociocultural (SO—purple), and recreational (RE—yellow). Some tokens are translated into English (Supporting Information Table A2).

The aspect nouns most associated with positive adjectives were “cachoeir” (waterfall) with 20% of interactions, followed by “natur” (nature), “access” (access), and “trilh” (trail), all with 8% of interactions. The aspect nouns most associated with negative adjectives were “estrad” (road) with 16% of interactions, followed by “acess” (access) with 14% of interactions and “trilh” (trail) with 12% of interactions. With regard to the qualifier adjectives, the most frequent positive ones were “lind” (beautiful) with 20% of interactions, followed by “maravilh” (wonderful) and “otim” (great), both with 13% of interactions. Conversely, the most frequent negative adjectives were “dificil” (difficult) with 26% of interactions, followed by “ruim” (bad) with 21% of interactions, and “cansat” (tiring) and “distant” (distant), both with 13% of interactions (Figure 6). The analysis also revealed a substantial interaction between esthetic aspects and positive adjectives, as well as between structural aspects and negative adjectives (Figure 6).

## 4 Discussion

A predominance of PA aspects associated with positive qualifiers emerged from our analysis, reflecting the prominent role of esthetic dimensions in shaping tourists' perceptions of nature. This pattern suggests that visitors value the visual and experiential qualities of protected landscapes, emphasizing that tourists benefit from ecosystem services through their interactions with nature. Our findings highlight the substantial role of esthetic elements in shaping tourists' perceptions (Campos and Filetto 2011; Ferreira et al. 2020). Notably, the higher representation of words related to the esthetic aspect and the lower prevalence of words from this category associated with negative adjectives, beyond what was expected by chance, further reinforce the importance of esthetics in tourist comments. This suggests that visitors to PAs seek a positive connection with nature, often engaging with natural attractions such as waterfalls, peaks, and caves. The PAs in the state of Minas Gerais, as revealed by our study, play a pivotal role in facilitating positive human–nature interactions. Such engagement contributes to the physical, psychological, and social well‐being of tourists (Jiricka‐Pürrer et al. 2019; Puhakka et al. 2017; Russell et al. 2013). Places with higher environmental quality, such as PAs, may also promote psychological recovery and foster a strong environmental connection in individuals (Wyles et al. 2019). Ultimately, our findings emphasize that the conservation of these areas extends beyond biodiversity preservation, playing a vital role in providing individuals with the opportunity to experience and connect with preserved nature, thereby contributing to both ecological and human well‐being.

Following the esthetic aspects of the PAs, the structural aspect of the PAs appears to play a significant role in shaping tourists' experiences, being frequently associated with positive qualifiers. The structural aspects, encompassing elements such as trails, access roads, and restaurants, are recognized as important factors that can influence how tourists engage with the CES provided by protected areas. However, negative perceptions of structural aspects were commonly reported by visitors to the protected areas. This finding suggests that negative perceptions related to structural aspects of PAs—such as poor infrastructure or inadequate signage—may impact the overall tourism experience, influencing how visitors connect with nature and perceive the ecosystem services offered by these areas (Kil et al. 2012; Soga et al. 2016; Zhang et al. 2014). Therefore, it is essential that managers ensure protected areas provide adequate infrastructure—such as clear trail signage, well‐maintained trails, and visitor facilities—that support tourist engagement without undermining conservation objectives. While it is essential to have well‐developed infrastructures to welcome tourists, it is important to ensure that the expansion of these structures does not compromise the natural aspects and conservation objectives of PAs.

Comments related to the socio‐cultural and recreational aspects of PAs appear to be less frequently mentioned by visitors. This observation may be attributed to the inherent variation in these aspects, such as museums, churches, mountain biking, and horseback riding, among PAs, which makes them less frequent overall. The limited representation, particularly of cultural aspects like tradition, crafts, and gastronomy, might create challenges by disconnecting PAs from local culture and community. The absence of emphasis on cultural elements may lead to the dissociation of PAs from the local culture, potentially impacting how the local community perceives these protected areas. Neglecting local culture can have repercussions, including increased stress and deteriorating mental health among the local population (Snodgrass et al. 2016). Moreover, the establishment of PAs that severely limit human activities, including traditional practices of the local community, could amplify negative perceptions and contribute to the unauthorized exploitation of resources (Alkan et al. 2009). This complexity exacerbates conservation efforts within PAs and intensifies conflicts between local communities and management authorities. Careful consideration of these socio‐cultural dynamics is essential for effective PA management and community engagement. In addition, tourists expressed more positive feelings in their comments, such as “tranquil,” “fascinating,” and “relaxing,” indicating that visitors find contact with nature evokes positive sensations (Ladeira et al. 2007). These emotions underscore the connection people establish with ecosystems, constituting what is termed a “sense of place,” recognized as a cultural ecosystem service (Hausmann et al. 2016; Ryfield et al. 2019). In conservation science, a sense of place has been linked to increased support for conservation efforts, as individuals who feel connected to nature are more inclined to advocate for its preservation (Lokhorst et al. 2014). The auditory environment, or soundscapes, also contributes to a sense of place, encompassing nature sounds, cultural‐historical sounds, and natural quiet soundscapes, which are acknowledged as vital for wildlife protection (Dumyahn and Pijanowski 2011). Studies have indicated that prolonged exposure to nature enhances the connection with the environment at any age (Cleary et al. 2020). Additionally, childhood experiences in nature have been shown to influence individuals positively, fostering environmentally conscious behaviors in adulthood (Wells and Lekies 2006). Thus, fostering contact with nature becomes crucial for strengthening the sense of place and garnering support for the conservation of protected areas. One potential approach for managers is to promote lower‐impact events that can foster greater contact with nature, such as birdwatching excursions, which tend to attract more ecologically aware ecotourists and can enhance knowledge of local birds (Sekercioglu 2002). Nevertheless, it is imperative to closely monitor tourist visitation to prevent uncontrolled and careless increases that may negatively impact the natural environment and wildlife (Carter et al. 2015; George and Crooks 2006; Ruschmann 1993) while diminishing the positive effect experienced by tourists (Taff et al. 2019).

We recognize certain limitations in our study. Social media platforms, including TripAdvisor, are more commonly used by younger individuals (Hausmann et al. 2018), and there is a tendency for internet users to predominantly express positive sentiments, despite potential underlying negative sentiments (Valdivia et al. 2019). Recognizing the limitations inherent in big data analysis, such as potential bias in representation among PA users and varying data availability for different areas, we note the advantages, including cost‐effectiveness and efficiency in analyzing a large volume of data (Wilkins et al. 2021). Furthermore, this approach can be complemented with traditional techniques, such as conducting workshops to gather people's opinions (Lee et al. 2020), comparing internet data with questionnaire responses (Hausmann et al. 2018), and incorporating official visitation statistics for parks (Tenkanen et al. 2017). Such an integrated approach allows for a comprehensive understanding of tourists' perceptions and can enhance the robustness of conservation and management strategies for PAs.

Despite these limitations, our findings can offer valuable guidance for PA managers by clarifying tourist preferences and underlining the importance of both contact with nature and well‐structured facilities. This insight can inform the development of effective marketing strategies aligned with conservation goals, such as promoting sustainable recreational and cultural experiences that engage visitors, local communities, and management authorities. Our analysis also supports the potential for customized assessments tailored to individual protected areas. By outlining each PA's unique features—highlighting both strengths and weaknesses—managers can derive targeted improvements in management, interpretation, and promotion (Hausmann et al. 2016). For example, if a biome is less visited but ecologically vital, managers could design experiences or communication specifically that showcase its unique natural values—thus enhancing its conservation appeal without degrading its ecological integrity.

## Author Contributions

**Marcela de Frias Barreto:** conceptualization (equal), data curation (equal), investigation (equal), methodology (equal), validation (equal), writing – original draft (equal). **Rodrigo Lima Massara:** conceptualization (equal), validation (equal), writing – review and editing (equal). **Arthur Soares Fernandes:** data curation (equal), formal analysis (equal), methodology (equal), validation (equal), writing – review and editing (equal). **Adriano Pereira Paglia:** conceptualization (equal), supervision (equal), writing – review and editing (equal).

## Conflicts of Interest

The authors declare no conflicts of interest.

## Supporting information


**Data S1:** Supporting Information.

## Data Availability

The data that supports the findings of this study is available in the Supporting Information of this article.
