# Supplementary material for: The Perception of Cultural Ecosystem Services by Tourists in Brazilian Protected Areas
Source: Ecol Evol. 2025 Nov 14;15(11):e72469. doi: 10.1002/ece3.72469 (PMC12617270; doi:10.1002/ece3.72469)
Supplement: Supplementary file 1 — Data S1: Supporting Information. [file ECE3-15-e72469-s001.docx]

**Supplementary material**

Table A1- List of protected areas used in the study, with their respective categories in the Brazilian Conservation Unit system, the name of the protected area, the number of comments on TripAdvisor, the number of visitors in 2016, type of vegetation in the Protected Area and who is responsible for managing the area (State, Federation or Private Property).

| Categories | Name | Number of comments | Visit (2016) | Vegetation | Management |
| --- | --- | --- | --- | --- | --- |
| Park | Itatiaia | 1322 | 127494 | Atlantic Forest | Federal |
| Park | Ibitipoca | 917 | 88837 | Atlantic Forest | State |
| Park | Serra do Cipó | 762 | 63871 | Cerrado | Federation |
| RPPN | Santuário do Caraça | 701 | - | Cerrado/ Atlantic Forest | Private Property |
| Park | Biribiri | 616 | 54621 | Cerrado | State |
| Park | Serra da Canastra | 454 | 68337 | Cerrado | Federation |
| Park | Serra do Rola Moça | 385 | 33656 | Cerrado/ Atlantic Forest | State |
| Park | Caparaó | 352 | 49617 | Atlantic Forest | Federation |
| Park | Itacolomi | 250 | 21419 | Atlantic Forest | State |
| Natural Monument | Gruta Rei do Mato | 224 | 19599 | Cerrado | State |
| Park | Cavernas do Peruaçu | 99 | 3929 | Cerrado/  Caatinga | Federation |
| Park | Serra do Ouro Branco | 86 | - | Atlantic Forest | State |
| Park | Nova Baden | 86 | 6312 | Atlantic Forest | State |
| Park | Rio Preto | 85 | 7602 | Cerrado | State |
| Park | Rio Doce | 74 | 13707 | Atlantic Forest | State |
| Park | Lapa Grande | 69 | 7037 | Cerrado | State |
| Park | Mata do Limoeiro | 68 | 27312 | Atlantic Forest/Cerrado | State |
| Park | Sumidouro | 53 | 34158 | Atlantic Forest/Cerrado | State |
| Park | Serra da Boa Esperança | 33 | - | Cerrado | State |
| Park | Pico do Itambé | 27 | 5682 | Atlantic Forest/Cerrado | State |
| Park | Serra do Brigadeiro | 25 | 7226 | Atlantic Forest | State |
| Park | Grão Mogol | 20 | - | Cerrado | State |
| Park | Serra do Gandarela | 16 | 0 | Atlantic Forest | Federation |
| Park | Serra do Intendente | 12 | 3824 | Atlantic Forest/ Cerrado | State |
| Park | Sete Salões | 11 | - | Atlantic Forest | State |
| RPPN | Feliciano Miguel Abdala | 4 | - | Atlantic Forest | Private Property |
| Park | Grande Sertão Veredas | 3 | 154 | Cerrado | Federation |

*Table A2- List of the five most cited words in each category and their respective frequencies. The categories are aspects (structure (st), aesthetics (ea), sociocultural (so), recreational (re)), and qualifiers (positive (po) and negative (ne)). Additionally, the words are in descending order of their frequency in the comments, for each category. In brackets is an example of a word that represents the token.*

| Categories | Tokens | Frequency |
| --- | --- | --- |
| ST | Trilh (e.g., trail) | 2344 |
|  | Acess (e.g., access) | 780 |
|  | Estrad (e.g., road) | 687 |
|  | Entrad (e.g., entrance) | 583 |
|  | Carr (e.g., car) | 510 |
| AE | Cachoeir (e.g., waterfall) | 3300 |
|  | Natur (e.g., nature) | 1669 |
|  | Agu (e.g., water) | 813 |
|  | Vist (e.g., View) | 689 |
|  | Pra (e.g., small beach) | 634 |
| SO | Conhec (e.g., knowledge) | 1049 |
|  | Histor (history) | 367 |
|  | Mus (e.g., museum) | 319 |
|  | Santuário (e.g., sanctuary) | 292 |
|  | Cas (e.g., house) | 251 |
| LE | Caminh (e.g., walking) | 1138 |
|  | Passeio (e.g., tour) | 1026 |
|  | Banh (e.g., bath) | 265 |
|  | Fot (e.g., photo) | 194 |
|  | Biciclet (e.g., mountain bike) | 156 |
| PO | Lind (e.g., beautiful) | 1766 |
|  | Maravilh (e.g., wonderful) | 905 |
|  | Otim (e.g., great) | 792 |
|  | Bonito (e.g., pretty) | 696 |
|  | Belo (e.g., nice) | 599 |
| NE | Dificil (e.g., difficult) | 145 |
|  | Ruim (e.g., bad) | 90 |
|  | Cansat (e.g., tiring) | 77 |
|  | Distant (e.g., distant) | 64 |
|  | Car (e.g., expensive) | 59 |

Tabela A3 – List of all tokens in the study with their respective frequencies and the category in which they were classified. The categories are aspects (structure (st), aesthetics (ea), sociocultural (so), recreational (re)), and qualifiers (positive (po) and negative (ne)).

| Token | Frequence | Category |
| --- | --- | --- |
| cachoeir | 3300 | aesthetics |
| trilh | 2344 | structure |
| lind | 1766 | positive |
| natur | 1669 | aesthetics |
| caminh | 1138 | leisure |
| conhec | 1049 | sociocultural |
| passei | 1026 | leisure |
| maravilh | 905 | positive |
| agu | 813 | aesthetics |
| otim | 792 | positive |
| acess | 780 | structure |
| bonit | 696 | positive |
| vist | 689 | aesthetics |
| estrad | 687 | structure |
| pra | 634 | aesthetics |
| bel | 599 | positive |
| bom | 598 | positive |
| entrad | 583 | structure |
| pic | 513 | aesthetics |
| carr | 510 | structure |
| restaur | 498 | structure |
| excelent | 468 | positive |
| estrutur | 441 | structure |
| cidad | 437 | structure |
| grut | 437 | aesthetics |
| gui | 431 | structure |
| sinaliz | 407 | structure |
| facil | 403 | positive |
| melhor | 385 | positive |
| histor | 367 | sociocultural |
| mirant | 357 | aesthetics |
| tranquil | 354 | positive |
| mus | 319 | sociocultural |
| are | 313 | aesthetics |
| vil | 308 | structure |
| pont | 300 | structure |
| divers | 299 | aesthetics |
| santuari | 292 | sociocultural |
| incrivel | 290 | positive |
| limp | 272 | positive |
| banh | 265 | leisure |
| proxim | 264 | positive |
| ceu | 261 | aesthetics |
| mat | 258 | aesthetics |
| paisag | 254 | aesthetics |
| cas | 251 | sociocultural |
| sup | 244 | positive |
| montanh | 238 | aesthetics |
| exuber | 236 | positive |
| preserv | 224 | aesthetics |
| port | 222 | structure |
| flor | 220 | aesthetics |
| pous | 216 | structure |
| encant | 215 | positive |
| sol | 215 | aesthetics |
| faun | 199 | aesthetics |
| pedr | 199 | aesthetics |
| conserv | 196 | aesthetics |
| fot | 194 | leisure |
| banheir | 193 | structure |
| belissim | 185 | positive |
| interess | 182 | positive |
| fantast | 175 | positive |
| camping | 170 | structure |
| lag | 164 | aesthetics |
| delic | 162 | positive |
| deslumbr | 159 | positive |
| biciclet | 156 | leisure |
| almoc | 153 | structure |
| estacion | 153 | structure |
| funcionari | 152 | structure |
| lago | 147 | aesthetics |
| cavern | 146 | aesthetics |
| dificil | 145 | negative |
| espetacul | 144 | positive |
| ideal | 142 | positive |
| infraestrutur | 136 | structure |
| lob | 136 | aesthetics |
| nascent | 133 | aesthetics |
| acamp | 129 | structure |
| imperdivel | 128 | positive |
| pur | 126 | positive |
| legal | 121 | positive |
| pe | 120 | leisure |
| pe | 120 | leisure |
| qued | 118 | aesthetics |
| bik | 117 | leisure |
| atend | 116 | structure |
| lanch | 115 | structure |
| gostos | 112 | positive |
| relax | 111 | positive |
| rios | 107 | aesthetics |
| igrej | 104 | sociocultural |
| passar | 103 | aesthetics |
| guar | 101 | aesthetics |
| impression | 95 | positive |
| poc | 95 | aesthetics |
| cerr | 94 | aesthetics |
| piscin | 94 | aesthetics |
| ruim | 90 | negative |
| valor | 90 | structure |
| atenc | 88 | positive |
| especial | 88 | positive |
| lanchonet | 87 | structure |
| sensacional | 87 | positive |
| plac | 86 | structure |
| nov | 85 | positive |
| bacan | 82 | positive |
| import | 78 | positive |
| cansat | 77 | negative |
| cert | 77 | positive |
| gratuit | 77 | positive |
| aconcheg | 72 | positive |
| espec | 70 | aesthetics |
| canion | 69 | aesthetics |
| espac | 69 | positive |
| hospedag | 68 | structure |
| magic | 67 | positive |
| abrig | 65 | structure |
| magnif | 65 | positive |
| asfalt | 64 | structure |
| caval | 64 | leisure |
| distant | 64 | negative |
| observ | 62 | aesthetics |
| hotel | 61 | structure |
| macac | 61 | aesthetics |
| escal | 60 | leisure |
| fotograf | 60 | leisure |
| car | 59 | negative |
| seguranc | 59 | structure |
| calm | 57 | positive |
| bar | 56 | structure |
| refresc | 56 | positive |
| colegi | 55 | sociocultural |
| veicul | 55 | structure |
| ecolog | 54 | aesthetics |
| pintur | 54 | sociocultural |
| ric | 54 | positive |
| sed | 54 | structure |
| famos | 53 | positive |
| guard | 51 | structure |
| perig | 51 | negative |
| map | 50 | structure |
| camp | 49 | aesthetics |
| marc | 49 | sociocultural |
| visa | 47 | aesthetics |
| d'agu | 46 | aesthetics |
| nativ | 46 | aesthetics |
| prainh | 46 | aesthetics |
| surpreendent | 46 | positive |
| viv | 45 | aesthetics |
| fabric | 44 | sociocultural |
| mar | 44 | aesthetics |
| plant | 44 | aesthetics |
| trekking | 44 | leisure |
| mes | 43 | structure |
| simpat | 43 | positive |
| pia | 42 | structure |
| corr | 41 | leisure |
| laz | 41 | leisure |
| estud | 40 | sociocultural |
| explic | 40 | sociocultural |
| aliment | 39 | structure |
| educ | 39 | sociocultural |
| quart | 39 | structure |
| escad | 38 | structure |
| servic | 38 | structure |
| vilarej | 38 | structure |
| aloj | 37 | structure |
| revigor | 37 | positive |
| mergulh | 36 | leisure |
| queij | 36 | sociocultural |
| sabor | 36 | positive |
| esport | 35 | leisure |
| florest | 35 | aesthetics |
| apaixon | 33 | positive |
| arvor | 33 | aesthetics |
| cultur | 33 | sociocultural |
| monitor | 33 | structure |
| pedal | 32 | leisure |
| recompens | 32 | positive |
| limpez | 31 | structure |
| travess | 31 | leisure |
| prestat | 30 | positive |
| silvestr | 30 | aesthetics |
| chal | 29 | structure |
| piqueniqu | 29 | leisure |
| sal | 29 | structure |
| cascat | 28 | aesthetics |
| real | 27 | positive |
| riach | 27 | aesthetics |
| roch | 27 | aesthetics |
| administr | 26 | structure |
| barat | 26 | positive |
| chuveir | 26 | structure |
| jip | 26 | leisure |
| churrasc | 25 | leisure |
| estalagmit | 25 | aesthetics |
| onibus | 25 | structure |
| suficient | 25 | positive |
| acolhedor | 24 | positive |
| bebedour | 24 | structure |
| charmos | 24 | positive |
| sinal | 24 | structure |
| arquitetur | 23 | sociocultural |
| formaca | 23 | aesthetics |
| intoc | 23 | positive |
| estalactit | 22 | aesthetics |
| seminari | 22 | sociocultural |
| vead | 22 | aesthetics |
| capel | 20 | sociocultural |
| divin | 20 | positive |
| post | 20 | structure |
| tamandu | 20 | aesthetics |
| avis | 19 | structure |
| biodivers | 19 | aesthetics |
| estont | 19 | positive |
| fascin | 19 | positive |
| loj | 19 | structure |
| miss | 19 | sociocultural |
| registr | 19 | sociocultural |
| abund | 18 | positive |
| anta | 18 | aesthetics |
| esquil | 18 | aesthetics |
| foga | 18 | structure |
| jog | 18 | leisure |
| negat | 18 | negative |
| pesquis | 18 | sociocultural |
| predi | 18 | sociocultural |
| rodov | 18 | structure |
| geolog | 17 | aesthetics |
| grandios | 17 | positive |
| inspir | 17 | positive |
| bibliotec | 16 | sociocultural |
| confort | 16 | positive |
| convid | 16 | positive |
| emocion | 16 | positive |
| medit | 16 | sociocultural |
| velh | 16 | negative |
| acerv | 15 | sociocultural |
| agrad | 15 | positive |
| carta | 15 | structure |
| escol | 15 | sociocultural |
| gastronom | 15 | sociocultural |
| grat | 15 | positive |
| passarel | 15 | structure |
| relev | 15 | aesthetics |
| sit | 15 | structure |
| som | 15 | aesthetics |
| tv | 15 | structure |
| vag | 15 | structure |
| cam | 14 | structure |
| churrasqueir | 14 | structure |
| cum | 14 | aesthetics |
| espiritual | 14 | sociocultural |
| esplendor | 14 | positive |
| hospitaleir | 14 | positive |
| imponent | 14 | positive |
| maquet | 14 | structure |
| pist | 14 | structure |
| biom | 13 | aesthetics |
| internet | 13 | structure |
| suj | 13 | negative |
| aul | 12 | sociocultural |
| bich | 12 | aesthetics |
| borbolet | 12 | aesthetics |
| fart | 12 | positive |
| mau | 12 | negative |
| paradisiac | 12 | positive |
| por-do-sol | 12 | aesthetics |
| precari | 12 | negative |
| rua | 12 | structure |
| correg | 11 | aesthetics |
| desgast | 11 | negative |
| dign | 11 | positive |
| event | 11 | leisure |
| plen | 11 | positive |
| rapel | 11 | leisure |
| religios | 11 | sociocultural |
| arte | 10 | sociocultural |
| divulg | 10 | structure |
| esplend | 10 | positive |
| feliz | 10 | positive |
| quadr | 10 | sociocultural |
| quiosqu | 10 | structure |
| refeica | 10 | structure |
| religia | 10 | sociocultural |
| solicit | 10 | positive |
| trist | 10 | negative |
| alegr | 9 | positive |
| corredeir | 9 | aesthetics |
| corrima | 9 | structure |
| espeleotem | 9 | aesthetics |
| gal | 9 | aesthetics |
| lugarej | 9 | structure |
| majest | 9 | positive |
| pareda | 9 | aesthetics |
| posit | 9 | positive |
| preg | 9 | aesthetics |
| ruin | 9 | sociocultural |
| tour | 9 | leisure |
| vinh | 9 | sociocultural |
| artesanat | 8 | sociocultural |
| competent | 8 | positive |
| eficient | 8 | positive |
| excursa | 8 | leisure |
| extraordinari | 8 | positive |
| fe | 8 | sociocultural |
| font | 8 | aesthetics |
| horrivel | 8 | negative |
| mic | 8 | aesthetics |
| mosteir | 8 | sociocultural |
| orquid | 8 | aesthetics |
| pagament | 8 | structure |
| prazer | 8 | positive |
| tecelag | 8 | sociocultural |
| tucan | 8 | aesthetics |
| alun | 7 | sociocultural |
| animal | 7 | aesthetics |
| atraent | 7 | positive |
| auditori | 7 | structure |
| barc | 7 | leisure |
| cang | 7 | aesthetics |
| capelinh | 7 | sociocultural |
| ema | 7 | aesthetics |
| industr | 7 | sociocultural |
| inset | 7 | aesthetics |
| marcant | 7 | positive |
| mosquit | 7 | aesthetics |
| peix | 7 | aesthetics |
| porteir | 7 | structure |
| van | 7 | structure |
| bromel | 6 | aesthetics |
| cantin | 6 | structure |
| carrapat | 6 | aesthetics |
| casari | 6 | sociocultural |
| cordial | 6 | positive |
| ecossistem | 6 | aesthetics |
| gentil | 6 | positive |
| ilumin | 6 | structure |
| salt | 6 | leisure |
| tens | 6 | negative |
| acessibil | 5 | structure |
| buffet | 5 | structure |
| crimin | 5 | negative |
| duch | 5 | structure |
| escultur | 5 | aesthetics |
| extinca | 5 | aesthetics |
| fabul | 5 | positive |
| frac | 5 | negative |
| guarit | 5 | structure |
| instal | 5 | structure |
| lua | 5 | aesthetics |
| planalt | 5 | aesthetics |
| produt | 5 | sociocultural |
| refeitori | 5 | structure |
| sala | 5 | aesthetics |
| sempre-v | 5 | aesthetics |
| vig | 5 | structure |
| confus | 4 | negative |
| exercici | 4 | leisure |
| favorit | 4 | positive |
| fenomenal | 4 | positive |
| gesta | 4 | structure |
| insegur | 4 | negative |
| lend | 4 | sociocultural |
| porta | 4 | structure |
| gratific | 3 | positive |
| pizz | 3 | structure |
| tradica | 3 | sociocultural |
| util | 2 | positive |
| veget | 2 | aesthetics |
| acomod | 1 | structure |
| impec | 1 | positive |
| instruca | 1 | structure |
